# Acyl carrier protein tag can enhance tobacco etch virus protease stability and promote its covalent immobilisation

**DOI:** 10.1007/s00253-023-12377-8

**Published:** 2023-02-10

**Authors:** Xuefeng Li, Jiahua Huang, Junjie Zhou, Changsheng Sun, Yujiao Zheng, Yuan Wang, Jin Zhu, Shengbin Wang

**Affiliations:** 1 College of Life Sciences, South China Agricultural University, Guangzhou, 510642 People’s Republic of China; 2 Guangdong Provincial Key Laboratory of Protein Function and Regulation in Agricultural Organisms, Guangzhou, 510642 People’s Republic of China

**Keywords:** TEV, ACP, Covalent immobilisation, Fusion protein production, Tag removal

## Abstract

**Abstract:**

Fusion expression is widely employed to enhance the solubility of recombinant proteins. However, removal of the fusion tag is often required due to its potential impact on the structure and activity of passenger proteins. Tobacco etch virus (TEV) protease is widely used for this purpose due to its stringent sequence recognition. In the present work, fusion to the acyl carrier protein from *E. coli* fatty acid synthase (ACP) significantly increased the yield of recombinant soluble TEV, and the ACP tag also greatly improved TEV stability. The cleavage activity of TEV was not affected by the ACP fusion tag, and ACP-TEV retained high activity, even at unfavourable pH values. Moreover, ACP-TEV could be efficiently modified by co-expressed *E. coli* holo-ACP synthase (AcpS), leading to covalent attachment of 4′-phosphopantetheine (4′-PP) group to ACP. The sulfhydryl group of the long, flexible 4′-PP chain displayed high specific reactivity with iodoacetyl groups on the solid support. Thus, TEV could be immobilised effectively and conveniently via the active holo-ACP, and immobilised TEV retained high cleavage activity after a long storage period and several cycles of reuse. As a low-cost and recyclable biocatalyst, TEV immobilised by this method holds promise for biotechnological research and development.

**Key points:**

• *The ACP tag greatly increased the soluble expression and stability of TEV protease*.

• *The ACP tag did not affect the cleavage activity of TEV*.

• *The holo-ACP Tag effectively mediated the covalent immobilisation of TEV*.

## Introduction

Much effort has been made to improve the solubility of recombinant proteins (Costa et al. 2014). Most previous work in this field has focused on the discovery, development and refinement of solubility-enhancing fusion tags (Ki and Pack 2020). They are also widely utilised to mask the cellular toxicity of recombinant peptides (Li 2011a). A large variety of solubility enhancer tags have been identified. MBP is a large (43 kDa) periplasmic protein in *Escherichia coli*. When fused with a partner, MBP promotes the solubility of the target protein through its intrinsic chaperone activity (Bach et al. 2001). This passive role of MBP depends on the large hydrophobic area exposed on its surface (Nallamsetty and Waugh 2007). Similarly, SUMO improves the folding and solubility of its fusion partner by performing chaperone activities (Marblestone et al. 2006).

The formation of protein aggregates remains one of the major challenges in the development and commercialisation of successful protein-based products (Wang and Roberts 2018). Addition of fusion tags may bring about a new set of problems. Potential interference with the partner structure or its biological activities means that it is often wise to remove fusion tags. This process is normally implemented by enzymatic cleavage, in which site-specific proteases can be used under mild conditions (Li 2011b; Young et al. 2012). For large-scale protein production, the expense associated with the proteolytic removal of fusion tags may account for a large portion of the manufacturing cost. Furthermore, to purify target proteins, additional chromatography steps are often required to separate the fusion tags. Hence, tag removal often adds labour costs to recombinant protein production, and decreases productivity.

Immobilisation of proteins onto solid supports is of great importance in numerous applications (Cekaite et al. 2007; Marques et al. 2011; Phizicky et al. 2003). Immobilisation of enzymes allows enzyme preparations to be handled conveniently. Moreover, easier separation of enzyme from products makes downstream processing much more efficient and cost effective. Besides the advantages mentioned above, immobilisation often gives rise to improved enzyme performance through enhanced stability, increased activity or modulated selectivity. Site-specific covalent linkage allows target enzymes to be immobilised onto solid supports in a definite, controlled fashion with well-defined orientations (Meldal and Schoffelen 2016).

A productive strategy for covalent enzyme immobilisation is to utilise bioorthogonal chemistry. For a given protein, it is often difficult to select a conjugation site for incorporation of such groups. Chemistry-mediated immobilisation may also be difficult due to low stability and sensitivity of the protein of interest, which is often exposed to low pH, organic solvent, or a metal catalyst (Meldal and Schoffelen 2016). Development of an efficient covalent immobilisation method under mild conditions is therefore of practical significance.

Tobacco etch virus (TEV) protease performs excellently for both in vitro and in vivo cleavage of fusion proteins due to its high robustness and substrate specificity (Fang et al. 2013; Nallamsetty et al. 2004; Sun et al. 2012). Nowadays, TEV is widely exploited in biotechnology, industrial applications and in vivo cellular studies (Cesaratto et al. 2016). *E. coli* acyl carrier protein (ACP) involved in fatty acid synthesis is very acidic (isoelectric point = 4.1), and extremely soluble (> 40 mg/ml) (Cronan and Thomas 2009). Herein, we report that, as a fusion tag, ACP can greatly increase the soluble expression of TEV, and effectively protect it from aggregation. Excitingly, ACP could also effectively mediate the covalent immobilisation of this protease. These excellent properties make ACP a promising fusion tag for future practical applications.

## Materials and methods

### Molecular cloning and construction of expression vectors

The ACP gene (Gene ID 944,805) was first amplified from *E. coli* (strain MG1655) genomic DNA using primer 1 (GGTCATCATCATCATCATCACGGTGGTAGCACTATCGAAGAACGC) and primer 2 (ATGAATTCCGCCTGGTGGCCGTTGATG), followed by primer 3 (TCGGATCCGAAAACCTGTACTTTCAGGGTCATCATCATCATCATC) and primer 2. The resulting amplicon was digested with *Bam*HI and *Eco*RI, and inserted into vector pET-28b-MBP-TEV-WHZ (Wang et al. 2015), generating the pET28b-MBP-ACP-TEV plasmid for expression of the ACP-TEV fusion protein with a His_6_-tag at its N-terminus. To add nine arginine residues at the C-terminus of recombinant TEV, the fusion gene *MBP-TEV* was first amplified with primer 4 (TAATACGACTCACTATAGGG) and primer 5 (ACGACCTCTTCGACGACCTCCAGCACCGTTCATCAGCTGGGTAGC), then with primer 4 and primer 6 (CATAAGCTTATCTTCGACGACCTCTTCGACGACCTCTTCGACGAC) using pET28b-MBP-TEV as template (Sun et al. 2012). The resulting amplicon was digested with *Nco*I and *Hin*dIII and inserted into vector pET-28b, generating the pET-28b-MBP-TEV-R9 plasmid for expression of TEV with a His_6_-tag at its N-terminus and nine consecutive arginine residues at its C-terminus. The pET28b-ACP-G2-Amy-Histag, pET30a-His_6_-MBP-BCCP, pET28b-MBP-TEV and pBAD34-AcpS constructs were generated in our laboratory previously for expression of the fusion protein ACP-Amy and MBP-BCCP, TEV and holo-(acyl carrier protein) synthase AcpS, respectively (Chen et al. 2012; Sun et al. 2012).

### Expression and purification of recombinant proteins

Expression vectors pET28b-MBP-TEV, pET28b-MBP-ACP-TEV, pET28b-MBP-TEV-R9, pET28b-ACP-G2-Amy-Histag and pET30a-His_6_-MBP-BCCP were separately transformed into *E. coli* BL21 (DE3), and cells were grown overnight in Luria broth (LB) medium supplemented with kanamycin (50 µg/ml). Cultures were then diluted 100-fold using the same medium and allowed to grow until they reached mid-log phase (optical density ~ 0.6–0.8 at 600 nm). Expression of recombinant TEV, ACP-TEV, TEV-R9 and MBP-BCCP was induced by adding isopropyl-β-D-thiogalactopyranoside (IPTG) to a final concentration of 0.3 mM, and culturing was continued at 30 °C for 12 h. Expression of the ACP-G2-Amy fusion protein was induced by the addition of IPTG to 0.4 mM, and culturing was continued at 20 °C. For co-expression of ACP-TEV with AcpS, cells harbouring the pBAD34-AcpS and pET-28b-MBP-ACP-TEV constructs were induced by addition of 0.2 mM of IPTG and 0.2% arabinose.

A 10 ml sample of each culture was harvested by centrifugation at 5000* g* for 10 min and resuspended in 4 ml of lysis buffer (50 mM NaH_2_PO_4_/Na_2_HPO_4_, 0.3 M NaCl, 20 mM imidazole, pH 8.0), then lysed by sonication on ice. Lysates were centrifuged at 10,000* g* for 20 min, supernatants were recovered, and insoluble fractions were taken as inclusion bodies and dissolved in phosphate buffer (50 mM NaH_2_PO_4_/Na_2_HPO_4_, pH 7.0, 8 M urea). Supernatants and inclusion bodies were further analysed by 12% sodium dodecyl sulphate polyacrylamide gel electrophoresis (SDS-PAGE).

Recombinant proteins (TEV, TEV-R9, ACP-TEV, ACP-G2-Amy and MBP-BCCP) were purified according to previous reports (Sun et al. 2012). Chromatography was performed using a HisTrap FF nickel affinity column (1 ml) according to the protocol specified by the manufacturer (GE Healthcare Bio-Sciences AB, Uppsala, Sweden). The column was equilibrated with lysis buffer, the supernatant was loaded, the column was washed three times with washing buffer (50 mM NaH_2_PO_4_/Na_2_HPO_4_, 0.3 M NaCl, 40 mM imidazole, pH 8.0), and the recombinant proteins were eluted with elution buffer (50 mM Na_2_HPO_4_/Na_2_HPO_4_, 0.3 M NaCl, 250 mM imidazole, pH 8.0).

### Comparative analysis of the activities of recombinant TEV, ACP-TEV and TEV-R9

For comparative analysis of the activities of recombinant TEV and its ACP-TEV fusion protein, the substrate MBP-BCCP was mixed with TEV and ACP-TEV at a molar ratio of 10:1, and incubated at 25 °C in standard reaction buffer (50 mM TRIS–HCl, 0.5 mM EDTA, 1 mM DDT, pH 8.0). Aliquots were removed for SDS-PAGE analysis at different time intervals. The substrate ACP-G2-Amy was also mixed with TEV, ACP-TEV and TEV-R9 at a molar ratio of 10:1 and incubated at 25 °C for 1 h at different pH values for site-specific cleavage. Generation of the product α-amylase (Amy) was analysed by SDS-PAGE.

### Determination of the kinetic parameters of enzyme TEV and ACP-TEV

The catalytic activities of enzyme TEV and ACP-TEV were also assayed by varying substrate (MBP-BCCP) concentrations, ranging from 5 to 40 μM. The *K*_*m*_ and *V*_max_ were determined by fitting the data to the Michaelis–Menten equation using GraphPad Prism software. All assays were performed at least three times independently.

### Analysis of the protective roles of the ACP fusion tag in preventing TEV aggregation induced by heat

A series of 1 mg/ml solutions of recombinant TEV and its ACP-TEV fusion protein were incubated at 30 °C, 35 °C, 40 °C and 45 °C for 4 h. Soluble proteins contained in the supernatants and aggregates were fractionated by centrifugation and analysed by SDS-PAGE. Both ACP-TEV and TEV were also incubated at 35 °C, aliquots were removed at different time intervals, and soluble proteins contained in the supernatants and the aggregates were analysed by SDS-PAGE.

### Mutation of TEV and analysis of mutant activity

The mutants, C110S/C130S TEV (mTEV-1) and C110G/C130G TEV (mTEV-2), were constructed by site-specific mutagenesis according to a previous report (Matos et al. 2008). Plasmid pET-28b-MBP-ACP-TEV was used as template. Primer 7 (CAGCGTGAAGAGCGTATCTCCCTGGTTACTACTAACTTC) and primer 8 (GAAGTTAGTAGTAACCAGGGAGATACGCTCTTCACGCTG) were employed to convert Cys-110 to Ser in TEV; primer 9 (CTATGGTATCTGATACCAGCTCCACTTTCCCGAGCAGCGAC) and primer 10 (GTCGCTGCTCGGGAAAGTGGAGCTGGTATCAGATACCATAG) were employed to convert Cys-130 to Ser in TEV; primer 11 (CAGCGTGAAGAGCGTATCGGCCTGGTTACTACTAACTTC) and primer 12 (GAAGTTAGTAGTAACCAGGCCGATACGCTCTTCACGCTG) were employed to convert Cys-110 to Gly in TEV; and primer 13 (CTATGGTATCTGATACCAGCGGCACTTTCCCGAGCAGCGAC) and primer 14 (GTCGCTGCTCGGGAAAGTGCCGCTGGTATCAGATACCATAG) were employed to convert Cys-130 to Gly in TEV. All mutations were confirmed by DNA sequence analysis.

After purification, the substrate MBP-BCCP was mixed with TEV, ACP-TEV, ACP-mTEV-1 and ACP-mTEV-2 at a molar ratio of 5:1 and incubated at 25 °C for 4 h in standard reaction buffer (50 mM TRIS–HCl, 0.5 mM EDTA, 1 mM DDT, pH 8.0) for site-specific cleavage. Generation of the product MBP was analysed by SDS-PAGE.

### Modification of the ACP-TEV fusion protein by AcpS

ACP-TEV was co-expressed with AcpS in *E. coli* cells harbouring the vectors pBAD34-AcpS and pET-28b-MBP-ACP-TEV for 12 h (Chen et al. 2012). After sonication, CoA was added to the clarified cell lysate to a concentration of 1 mM. The resulting mixture was incubated at 37 °C for 4 h to implement phosphopantetheinylation of the fusion protein, apo-ACP-TEV. Subsequently, purified holo-ACP-TEV was desalted and analysed by matrix-assisted laser desorption/ionisation time of flight mass spectrometry (MALDI-TOF–MS) to verify the modification.

### Covalent immobilisation of TEV

After phosphopantetheinylation, holo-ACP-TEV was covalently immobilised onto SulfoLink Coupling Resin (Thermo Scientific) according to the manufacturer’s instructions. The resin was first pre-treated with blocking solution containing a mixture of amino acids (50 mM Glu, 50 mM Met, 50 mM Ser, 50 mM Trp, 50 mM Arg, 50 mM Tyr, 50 mM Lys, 50 mM His, pH 9.5). A 200 μl sample of resin was immersed in 1 ml of blocking solution and gently shaken for 2 h at 37 °C. This treatment was to block potential reactive groups on the support, which might react with groups on the protein surface, such as –COOH, –NH2 and –OH. The resin was then mixed with target proteins (~ 1.6 mg of ACP-TEV or TEV) contained in coupling buffer (1 mM EDTA, 50 mM TRIS–HCl, pH 8.8) and gently rocked at 37 °C for 2 h. The amount of target protein immobilised on the SulfoLink coupling resin was determined from the difference between the concentration of starting protein sample applied to the resin and that of the uncoupled fraction recovered. The remaining active iodoacetyl groups on the resin were blocked by addition of 1 ml of quenching buffer (5 mM EDTA, 50 mM TRIS–HCl, 50 mM Cys-HCl, pH 8.5). Immobilised proteins were stored in storage buffer (0.1 M NaH_2_PO_4_/Na_2_HPO_4_, 50% glycerol, 0.05% NaN_3_, pH 7.0) at − 20 °C.

To analyse the cleavage activity of immobilised TEV, 4 ml of substrate MBP-BCCP was mixed with 200 μl of resin on which TEV was immobilised. The protein sample was passed through the resin in the column at a continuous rate using a peristaltic pump at the rate of 100 μl/min. Aliquots were removed for SDS-PAGE analysis at different time intervals to detect the MBP product.

## Results

### Fusing to ACP increases the accumulation of soluble protease TEV

**Fig. 1:**
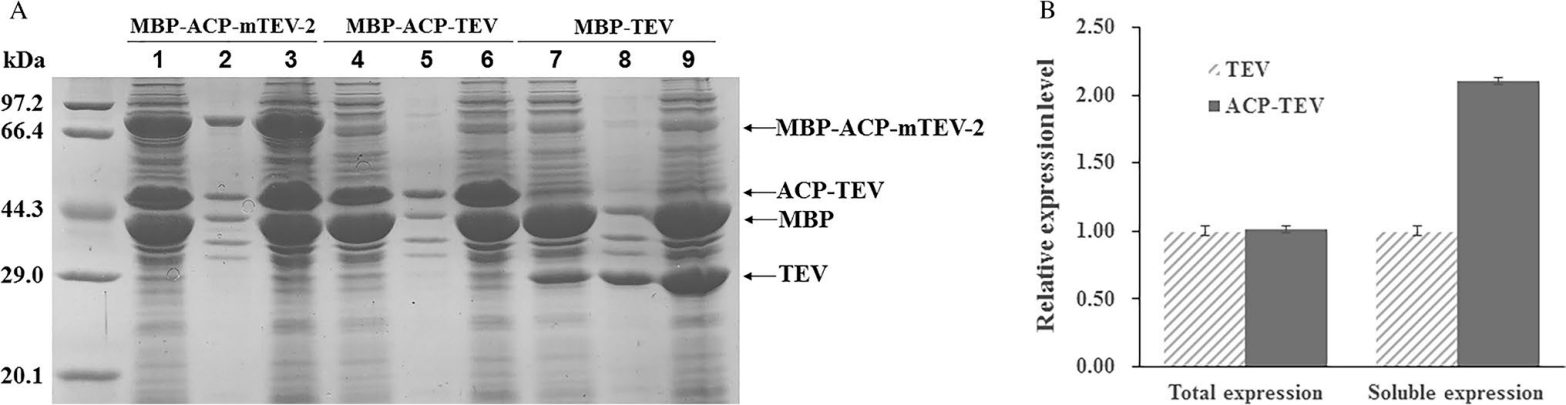
The ACP fusion tag increases soluble expression of the tobacco etch virus (TEV) protease passenger. After expressing recombinant proteins at 30 °C for 12 h, *E, coli* cells were harvested and lysed by sonication. Lysates were clarified by centrifugation and precipitates served as inclusion bodies. Total cell contents, inclusion bodies and supernatants were analysed by SDS-PAGE. Lane 1, lane 4 and lane 7: supernatants; lane 2, lane 5 and lane 8: inclusion bodies; lane 3, lane 6 and lane 9: total cell contents. **A** SDS–PAGE analysis of the expression of recombinant proteins. **B** The relative expression levels of recombinant TEV and ACP-TEV fusion protein, the expression level of recombinant TEV was set to 1

*E. coli* ACP is a small acidic protein of 77 amino acid residues (Cronan and Thomas 2009). Its excellent physicochemical characteristics prompted us to investigate its potential effect on the enhancement of soluble expression of TEV. We constructed the MBP-ACP-TEV fusion protein, in which a TEV recognition sequence (ENLYFQ) was inserted between MBP and ACP-TEV. MBP-ACP-TEV underwent efficient intracellular self-cleavage, generating the recombinant ACP-TEV fusion protein (Fig. 1). The efficient self-cleavage proved that ACP-TEV possesses high site-specific cleavage activity. Our results also demonstrated that as a fusion tag, ACP could significantly increase the soluble accumulation of TEV by ~ 100%.

We also created mutants, C110G/C130G TEV and C110S/C130S TEV, in which the Cys residue on the protein surface was mutated. As shown in Fig. 1, the C110G and C130G substitution resulted in a large accumulation of recombinant MBP-ACP-mTEV fusion proteins, implying that the resulting mutant could not self-cleave efficiently. Substitution of Ser for Cys-100 and Cys-130 on the TEV surface also brought about an obvious accumulation of the fusion protein MBP-ACP-mTEV (C110S/C130S) (data not shown). Our results suggest that the Cys residue on the TEV surface might play an important role in its activity.

### Fusing to ACP protects TEV from aggregation induced by heat

**Fig. 2:**
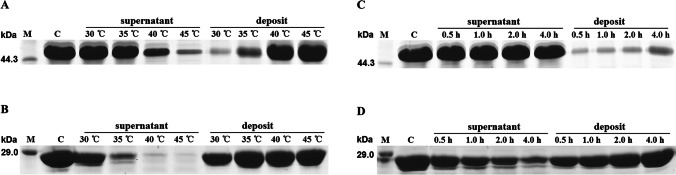
The ACP fusion tag prevents temperature-induced TEV aggregation. Recombinant ACP-TEV and its TEV counterpart were incubated at different temperatures for 4 h or at a defined temperature for different time intervals. Soluble proteins contained in the supernatants and insoluble aggregates were fractioned by centrifugation, then analysed by SDS-PAGE. **A** Aggregation of the ACP-TEV fusion protein induced at different temperatures. **B** Aggregation of TEV induced at different temperatures. **C** Formation of aggregates of the ACP-TEV fusion protein at different time intervals at 35 °C. **D** Formation of aggregates of TEV at different time intervals at 35 °C

Aggregation of proteins causes serious issues, such as a sharp reduction in activity during manufacturing processes or storage, and enhanced cytotoxicity and immunogenicity of pharmaceuticals. TEV retains high activity under a wide range of buffer conditions, and tolerates high concentrations of some detergents, denaturants and reducing agents (Sun et al. 2012). However, TEV is highly sensitive to elevated temperatures. The distinguishing characteristics of ACP prompted us to investigate its potential effect on the thermostability of TEV. When TEV was fused with ACP, the resulting ACP-TEV fusion protein became much more thermostable compared with TEV alone. Most of the TEV molecules aggregated after incubation at 35 °C for 4 h, and over half formed insoluble aggregates within 1 h (Fig. 2). By contrast, ACP-TEV was much more thermostable under the same temperature conditions, and no more than 20% of fusion protein molecules formed insoluble aggregates within 4 h at 35 °C (Fig. 2).

### Comparative analysis of ACP-TEV enzyme activity

Many fusion tags have been demonstrated to significantly enhance the soluble expression of recombinant proteins (Ki and Pack 2020). Removal of the fusion tag from a given fusion protein is often necessary due to its potential interference with the native structure and functioning of target proteins. Development of fusion tags that have no major impact on the biological activities of passenger proteins is of special significance. As shown in Fig. 1, when the fusion protein MBP-ACP-TEV was expressed, self-cleavage occurred efficiently in vivo, leading to the generation of MBP and ACP-TEV. These results indicated that the ACP-TEV fusion protein possessed high activity, similar to that of TEV alone, and that the ACP fusion tag barely affected the biological activity of the TEV passenger in this context.

**Fig. 3:**
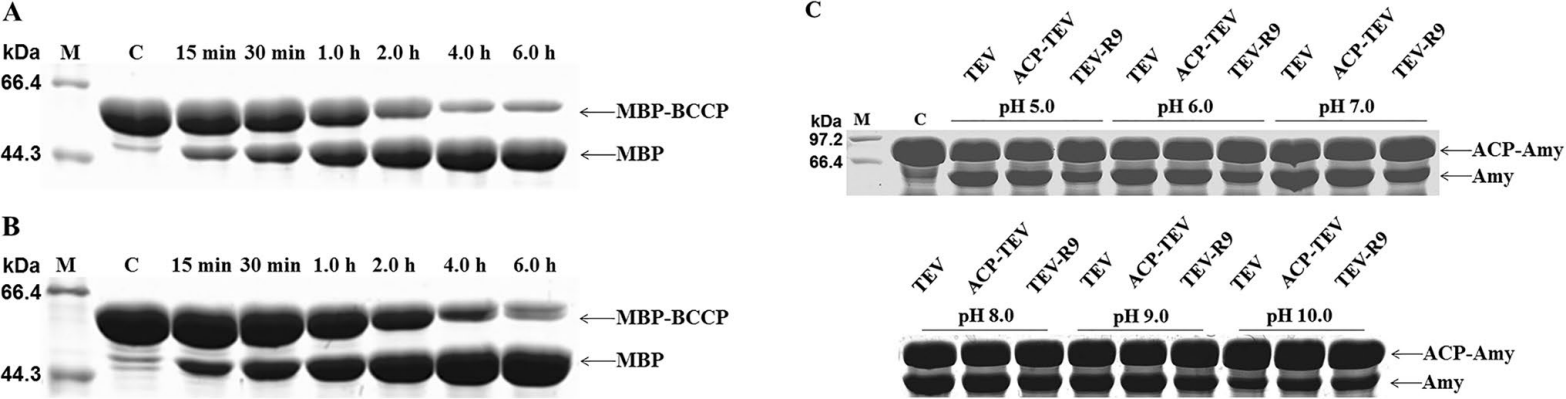
Comparative analysis of the cleavage activity of TEV and the ACP-TEV fusion protein under different conditions. **A** Evaluation of the cleavage activity of TEV under standard buffer conditions. **B** Evaluation of the cleavage activity of ACP-TEV under standard buffer conditions. **C** A specific recognition site (ENLYFQ) for TEV was inserted between the ACP fusion tag and the α-amylase (Amy) passenger protein. ACP-Amy was subjected to site-specific cleavage by TEV, ACP-TEV and TEV-R9 for 1 h at a molar ratio of 10:1 at different pH values. Generation of the Amy product was analysed by SDS-PAGE

We compared the cleavage activity of ACP-TEV with that of TEV in vitro. When the same amount of MBP-BCCP fusion protein was separately subjected to enzymatic cleavage by ACP-TEV and TEV, an almost equal amount of MBP product was produced at different time intervals (Fig. 3A, B). Our results clearly demonstrated that ACP-TEV exhibited the same enzymatic activity as TEV, suggesting that the ACP fusion tag had no negative effect on the cleavage activity of the TEV passenger protein.

We then investigated the potential roles of ACP in maintaining the enzymatic activity of its TEV passenger protein at different pH values. As shown in Fig. 3C, ACP-TEV retained high cleavage activity over a broad pH range, and exhibited higher enzymatic activity than TEV alone under alkaline conditions (pH 10.0), reinforcing the superior performance of ACP as a fusion tag.

We also investigated the kinetic characteristics of TEV and ACP-TEV. The *K*_m_ values of an enzyme suggest the affinity of an enzyme for its substrates. Under our experimental conditions, the *K*_m_ values for ACP-TEV were smaller than those for TEV (Table 1). The slight decrease in the *K*_m_ value of the ACP fusion construct implied that the tag ACP could help to maintain the stability or the native conformation of the enzyme TEV, bringing about relatively higher affinity for its substrates. Another important parameter for a given enzymatic reaction is *k*_cat_/*K*_m_, reflecting the catalytic efficiency. As shown in Table 1, the *k*_cat_/*K*_m_ value of fusion enzyme ACP-TEV was very close to that of TEV. Our data showed that linkage of the fusion tag ACP had no negative effects on the catalytic efficiency of TEV.

**Table 1:** Kinetic parameters of enzyme TEV and ACP-TEV

Enzyme	*K*_*m*_ (μM)	*k*_cat_ (min^−1^)	*k*_cat_/*K*_*m*_ (μM^−1^ min^−1^)
TEV	2.56 ± 0.26	313.86 × 10^−3^ ± 28.25 × 10^−3^	122.60 × 10^−3^ ± 11.28 × 10^−3^
ACP-TEV	1.57 ± 0.16	176.55 × 10^−3^ ± 18.19 × 10^−3^	112.45 × 10^−3^ ± 10.36 × 10^−3^

### Fusing to ACP results in effective covalent immobilisation of TEV

**Fig. 4:**
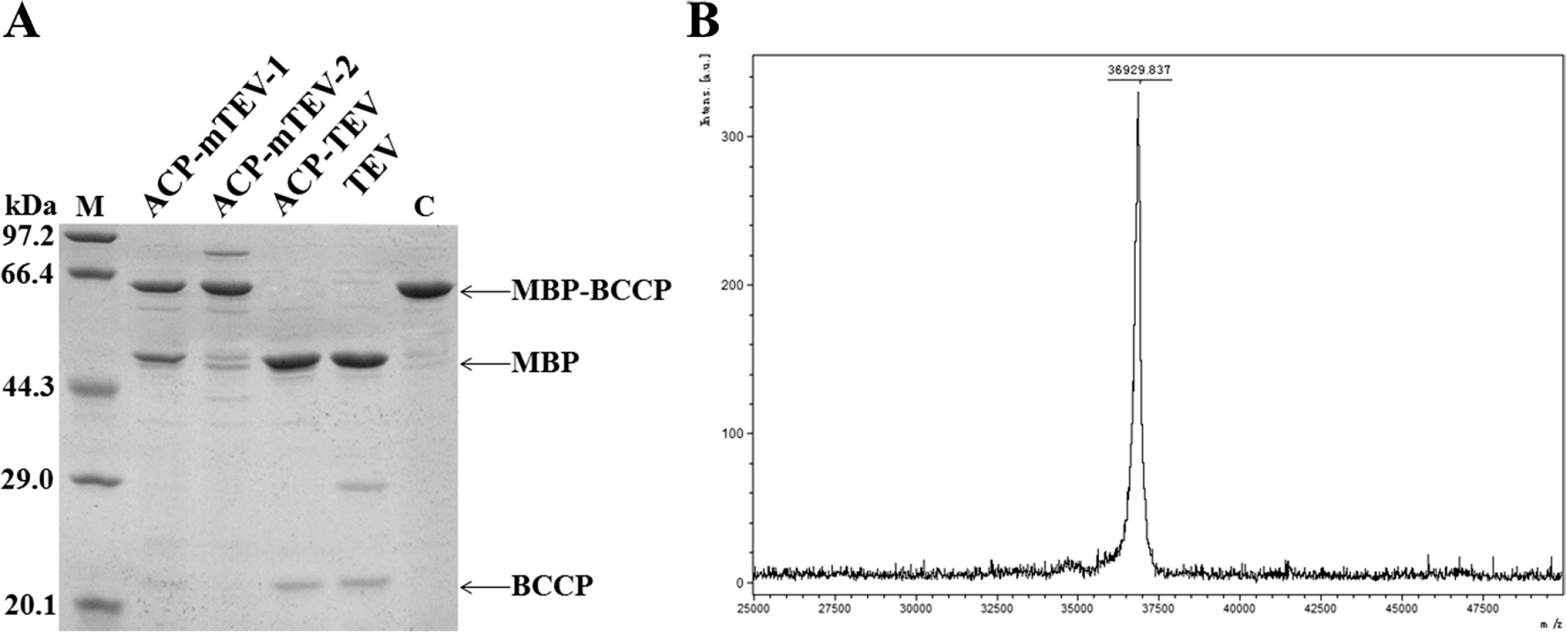
**A** Effects of mutation of cysteine residues on the cleavage activity of TEV. The fusion protein MBP-BCCP was subjected to site-specific cleavage by ACP-mTEV-1, ACP-mTEV-2, ACP-TEV and TEV for 4 h at a molar ratio of 5:1. Generation of the MBP product was analysed by SDS-PAGE. ACP-mTEV-1: two cysteine residues on the TEV surface were replaced by serine; ACP-mTEV-2: two cysteine residues on the TEV surface were replaced by glycine. **B** MALDI-TOF mass spectral analysis of phosphopantetheinylation of the fusion protein ACP-TEV. The peak with mass 36,929.8 corresponds to the theoretical molecular weight of phosphopantetheinylated ACP-TEV (holo-ACP-TEV; 36947.59)

TEV is expensive when exploited to cleave fusion proteins at a large scale. Covalent immobilisation of TEV may solve this issue to some extent due to its reusability. Sulfhydryl groups readily react with iodoacetyl groups under mild basic conditions to form a stable carbon–sulphur linkage (Mallik et al. 2007). Thus, thiol groups of Cys residues on a given protein surface have the potential to mediate its immobilisation via specific covalent linkage to supports bearing thiol-reactive groups (Puhl et al. 2009). However, this specific reaction can result in uncontrolled immobilisation when more than one Cys residue is present on the protein surface. In *E. coli*, apo-ACP is posttranslationally modified and converted to active holo-ACP via covalent attachment of 4′-phosphopantetheine (4′-PP) from coenzyme A (CoA) by the enzyme holo-ACP synthase (AcpS) (Cronan and Thomas 2009). The 4′-PP moiety provides holo-ACP with a long, flexible arm, and the sulfhydryl group of 4′-PP is highly reactive. The results presented in Fig. 1 show that the Cys residues on the surface of TEV play important roles in cleavage activity. We further analysed the activities of mutants C110S/C130S TEV and C110G/C130G TEV. As anticipated, replacement of Cys with Ser or Gly seriously affected the cleavage activity of TEV (Fig. 4A), implying that the enzymatic activity of immobilised TEV would be affected if the Cys residue on the TEV surface is exploited for covalent linkage.

**Fig. 5:**
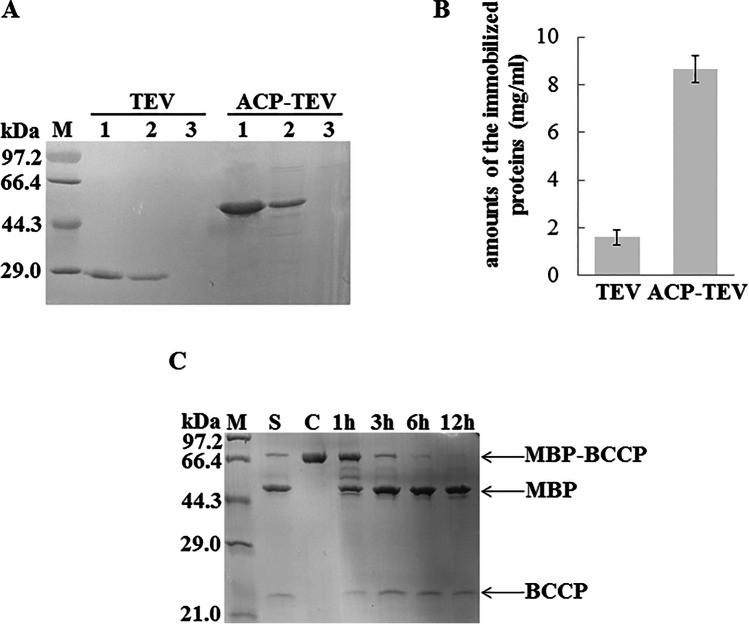
**A** Covalent immobilisation of holo-ACP-TEV and TEV via formation of a stable carbon–sulphur linkage between the sulfhydryl group and the iodoacetyl group under mild basic conditions. Lane 1: the starting sample applied to perform immobilisation. Lane 2: the free protein after immobilisation. Lane 3: washing buffer. **B** Analysis of the cleavage activity of immobilised TEV. Products were analysed after site-specific cleavage of the substrate MBP-BCCP by immobilised ACP-TEV for 1 h, 3 h, 6 h and 12 h. S: products generated from site-specific cleavage of the substrate MBP-BCCP by free TEV; **C** substrate MBP-BCCP

The characteristic properties of ACP were exploited to develop a novel method for immobilisation of TEV. In the ACP-TEV fusion protein, the TEV passenger may also influence the structure of ACP in reverse. The degree of phosphopantetheinylation of ACP-TEV was analysed by MALDI-TOF mass spectrometry after it was modified by AcpS. Excitingly, ACP-TEV could be modified efficiently; almost 100% of ACP-TEV molecules were modified (Fig. 4B). After being modified by AcpS, the resulting holo-ACP-TEV fusion protein was loaded onto a column containing coupling resin in mild buffer solution (50 mM TRIS–HCl, 5 mM EDTA-Na, pH8.5) at 37 °C. The amount of holo-ACP-TEV immobilised on the resin was ~ 8.64 mg per ml of resin, suggesting that the 4′-PP group covalently linked to ACP was highly reactive (Fig. 5A, B).

The TEV protein itself possesses two free thiol groups on its surface, allowing ~ 1.60 mg of TEV to be immobilised per ml of coupling resin. The amount of holo-ACP-TEV immobilised far exceeded that of TEV, demonstrating that the chemoselective reaction between the 4′-PP group and the support allowed the target protein to be immobilised with high efficiency (Fig. 5A, B). It is reasonable to speculate that the long, flexible 4′-PP group played a crucial role, allowing ACP-TEV to be immobilised in larger quantities. Our results clearly demonstrated that holo-ACP can function as a powerful tag to mediate the covalent immobilisation of TEV.

We next tested the enzymatic activity of immobilised ACP-TEV. As shown in Fig. 5C, TEV immobilised via holo-ACP could cleave the fusion tag efficiently, demonstrating that TEV immobilised by the method presented herein retained high cleavage activity. In our laboratory, immobilised TEV has already been widely used to cleave fusion proteins, and it can retain over 50% of its original activity after being stored at − 20 °C for 60 days and reutilised for at least five cycles of cleavage (data not shown).

## Discussion

Protein aggregation is generally regarded as a series of sequential and parallel processes (Bakou et al. 2017). In most cases, intrinsic factors are associated with internal structural changes in proteins, eventually leading to visible particles due to decreased solubility (Alam et al. 2017; Ow and Dunstan 2014). TEV typically forms inclusion bodies when overexpressed (van den Berg et al. 2006). Thus, it is often expressed in fusion form with an MBP tag, in which a TEV recognition sequence (ENLYFQ) is inserted between MBP and TEV (Sun et al. 2012). For highly charged fusion tags, the net charge is suggested to play a crucial role in preventing protein aggregation due to electrostatic repulsion, providing the target proteins with adequate time for further correct folding (Costa et al. 2014). In the *E. coli* cytosol, folding intermediates of nascent protein chains typically either adopt the correct conformation or appear in the form of inclusion bodies, and this balance determines the final accumulation levels of soluble and aggregated recombinant proteins. Compared with its TEV counterpart, a larger amount of soluble ACP-TEV accumulated in the present work. ACP is a very acidic protein. Thus, the high net charge of ACP confers its fusions with strong electrostatic repulsion, significantly reducing interactions between fusion proteins and subsequent oligomerisation, resulting in fewer insoluble particles. Nevertheless, we cannot exclude the possibility that the rapid folding properties and high solubility of ACP might allow it to function as a nucleation site for nursing the proper folding of TEV, or improving the reversibility of the aggregation process, resulting in increased soluble accumulation levels of the ACP-TEV fusion protein.

In most cases, fusion tags are removed from fused proteins by site-specific enzymatic cleavage, and the released passenger proteins are purified by a second purification step (Costa et al. 2014). Tag removal adds another layer of tedious labour and expense to the production of recombinant target proteins expressed in fusion form. Relatively few reports have characterised fusion tags that do not impair the biological activities of passenger proteins so that removal of the fusion tag can be avoided. Our data clearly showed that tag ACP barely affected the activity of its passenger protein TEV. Furthermore, even at 30 °C, over half of recombinant TEV molecules formed insoluble particles after incubation for 4 h in viro (Fig. 2), implying that TEV is sensitive to temperature. When TEV was fused with ACP, the dense positive charges on the ACP surface may help to protect ACP-TEV from aggregating via electrostatic repulsion, thereby enhancing the solubility. This may explain why ACP-TEV exhibited higher enzymatic activity than its TEV counterpart under unfavourable pH conditions (Fig. 3). This hypothesis was further confirmed by the similarly excellent performance of modified TEV (TEV-R9), which is highly positively charged (Fig. 3).

Compared with non-covalent immobilisation processes, site-specific covalent linkage not only allows target proteins to be immobilised onto solid supports more stably, but also in a more controlled fashion, which helps to address the orientation issue of attached proteins. Even though TEV can be immobilised via strong conjugation between the fusion tag streptavidin and a biotin-labelled solid support, gradual loss of the TEV-streptavidin fusion protein from the support was detected due to dissociation of the streptavidin–biotin complex over extended time intervals (Norris et al. 2020). Cysteine residues on the TEV surface have been exploited to mediate covalent immobilisation via the formation of disulfide bonds (Puhl et al. 2009). Regrettably, it was reported that this immobilisation process caused about 70% of the protease to be inactivated. Our current results also demonstrated that these cysteine residues played important roles in the cleavage activity of TEV. Direct covalent linkage between these cysteine residues and iodoacetyl groups on solid supports may create distortion in the TEV structure, resulting in the loss of its activity. Moreover, the possibility cannot be excluded that immobilisation mediated by cysteine residues on the TEV surface may cause the active site of TEV to orient toward the solid support, thereby limiting the accessibility of substrates and bringing about a decrease in its apparent activity. The development of efficient and convenient methods for immobilising TEV with high cleavage activity is therefore of great significance in light of its prospects in biotechnological research and development.

As a fusion tag, ACP not only improved the stability of TEV and preserved its high enzymatic activity, it also facilitated its efficient covalent immobilisation. In holo-ACP, the long, flexible 4′-PP group can act like a “spacer”. The use of a spacer is desirable because it creates distance between the support and the immobilised protein, which reduces the potential for denaturation and facilitates interaction between substrates and enzymes (Chakraborty et al. 2016). Furthermore, the thiol group of holo-ACP was highly reactive with iodoacetyl groups under very mild conditions, allowing immobilisation to be accomplished within about 1 h. It is reasonable to speculate that a combination of efficient production and effective immobilisation of TEV means that the method presented herein has great potential for applications in the future.

TEV has great potential in biotechnological research and development due to its stringent sequence recognition. However, TEV is expensive and prone to aggregation even under low temperature, resulting in an obvious loss in activity. In this study, we found that fusion to an ACP tag could improve the yield and thermostability of recombinant TEV significantly. Moreover, efficient phosphopantetheinylation of ACP-TEV could be achieved by simple co-expression of AcpS. The long, flexible 4′-PP group was able to confer efficient immobilisation of TEV under very mild conditions. It is reasonable to speculate that immobilised ACP-TEV will expand the usage of this site-specific protease for biotechnology.

## Data Availability

The datasets generated during and/or analysed during the current study are available from the corresponding author on reasonable request.
